# Perceived Diagnostic Value of Fluorescence-Enhanced 3D Imaging for Detecting Caries Adjacent to Restorations: A Questionnaire-Based Study

**DOI:** 10.3390/dj14010061

**Published:** 2026-01-16

**Authors:** Dimitrios Spagopoulos, Grigoria Gkavela, Christos Rahiotis

**Affiliations:** Department of Operative Dentistry, Dental School, National and Kapodistrian University of Athens, 11527 Athens, Greece

**Keywords:** fluorescence, caries adjacent to restorations, diagnostic value, questionnaire, digital dentistry

## Abstract

**Background/Objectives:** Caries adjacent to restorations remain a leading cause of restoration failure and replacement. Conventional diagnostic methods are limited by subjectivity and restricted visualization. Fluorescence-enhanced three-dimensional (3D) imaging has been proposed to improve detection accuracy, but evidence on its clinical perception and usability remains scarce. The objective of this study was to evaluate the perceived diagnostic value of fluorescence-enhanced 3D imaging in detecting caries adjacent to direct restorations. **Methods:** A cross-sectional questionnaire-based survey was distributed to undergraduate dental students and licensed dentists (n = 94). Participants assessed images of extracted teeth with direct restorations presented in three formats: conventional photographs, monochromatic 3D models, and 3D models with fluorescence. Responses were analyzed using descriptive statistics, chi-square tests, and Cohen’s kappa to measure inter-rater agreement. **Results**: Overall, 64.9% of respondents reported that fluorescence-enhanced images improved their diagnostic decision-making, while 29.8% reported partial benefit. Fluorescence was mainly perceived as helpful in defining cavity margins (53.3%) and assessing lesion volume (42.4%). Most participants preferred 3D models with fluorescence over conventional images for diagnostic value. However, inter-rater agreement was generally poor (κ range: –0.05 to 0.25; median κ = 0.02; only 4 images showed weak but statistically significant agreement), with only a few images demonstrating weak but statistically significant agreement. Notably, 39.3% of participants reported prior experience with 3D imaging, which was associated with greater confidence in interpreting fluorescence-enhanced images. Participants with prior 3D imaging experience reported greater confidence in fluorescence interpretation. **Conclusions**: While fluorescence-enhanced 3D imaging is perceived as a useful adjunct for visualizing lesion margins and depth, it does not currently yield consistent diagnostic agreement across clinicians. Training, calibration, and integration of artificial intelligence support may enhance the clinical reliability of this technology.

## 1. Introduction

Secondary caries or caries adjacent to restorations—defined as carious lesions that develop at the margins of existing restorations—remain one of the most frequent causes of restoration failure and replacement in clinical practice [1,2]. Despite advances in adhesive dentistry and restorative materials, accurately diagnosing marginal caries remains a clinical challenge. Early detection is essential for minimally invasive intervention; however, secondary lesions often go undetected until they have progressed significantly, compromising the restoration and the underlying tooth structure.

Traditional detection approaches, including visual–tactile inspection and bitewing radiography, are widely relied upon in daily clinical workflows. However, both techniques have well-defined limitations. Specifically, visual assessment is subject to examiner variability and depends heavily on illumination, dryness, magnification, and unobstructed access to the restoration margins. Recent studies [3,4,5,6] confirm that visibility factors—especially illumination, moisture control, and direct access—are more critical than examiner experience for accurately detecting marginal caries. Additionally, bitewing radiographs provide only two-dimensional information and may fail to reveal early marginal lesions due to projection geometry and superimposition of restorative materials and adjacent anatomical structures. These diagnostic constraints have been highlighted in recent comparative and methodological studies [6,7].

In response to these limitations, optical caries detection tools—particularly fluorescence-based systems—have emerged as promising adjuncts. These techniques exploit the differential light emission of demineralized enamel and bacterial metabolites when excited by specific wavelengths, offering enhanced visual contrast between healthy and carious tissues [8]. Several in vivo studies and systematic reviews [9,10] have demonstrated improved sensitivity of fluorescence tools, particularly for detecting occlusal and proximal lesions. However, most of these studies focus on primary caries and often neglect the complex diagnostic scenarios posed by restoration margins.

Parallel to this, intraoral scanners have revolutionized digital dentistry by enabling detailed three-dimensional (3D) visualizations of tooth morphology. The integration of fluorescence or optical reflectance data into 3D models has the potential to further enhance diagnostic clarity by providing spatial context and morphological accuracy, supporting better identification of marginal defects and lesion activity [10]. Such multimodal imaging could facilitate better identification of marginal defects and lesion activity, aiding in more precise clinical decision-making.

Despite a growing number of research articles on the detection of recurrent caries [10], existing studies are primarily limited to the technical validation of diagnostic tools in vitro or under controlled clinical conditions, focusing on sensitivity, specificity, and imaging properties. In contrast, the current study evaluates the subjective perception and usability of fluorescence-enhanced 3D imaging among dental students and clinicians, thereby expanding upon our previous systematic review of fluorescence and NIR for secondary caries detection [11]. This approach allows for the assessment of real-world clinician experience, perceived value, and potential implementation barriers. Most investigations focus on sensitivity, specificity, and imaging properties based on extracted teeth or in vitro models. Researchers typically conduct these studies, which focus on diagnostic technologies and do not involve clinicians, who are the end users of such tools. As a result, clinical usability and diagnostic confidence by dental professionals remain unexplored.

This gap highlights the rationale for conducting a targeted survey among dental practitioners (general or specialized) and students to assess how fluorescence-enhanced imaging is perceived and utilized in the diagnostic decision-making process. Understanding subjective experience, perceived value, and potential barriers to integration from a clinician’s perspective is essential for bridging the gap between technological development and everyday clinical use. By focusing on clinician experience, this study aims to generate insights that are not only academically relevant but also practically applicable in caries diagnostic workflows and educational strategies.

The aim of this study was to evaluate clinicians’ perceptions of fluorescence-enhanced 3D imaging as a diagnostic tool for detecting secondary caries at the margins of direct restorations. The primary objective was to assess the perceived diagnostic value of fluorescence-enhanced 3D imaging for identifying secondary caries at restoration margins. Secondary objectives included examining whether professional experience and prior familiarity with 3D imaging influenced diagnostic interpretation and clinician confidence, as well as evaluating the impact of visual access and visibility-related factors—such as illumination, moisture control, and retraction—on diagnostic performance. We hypothesized that fluorescence-enhanced 3D imaging would be perceived as a useful diagnostic adjunct; however, inter-rater agreement and diagnostic confidence would vary according to clinicians’ experience, prior exposure to 3D imaging, and the accessibility of the restoration margin.

## 2. Materials and Methods

### 2.1. Study Design

This study was a cross-sectional, questionnaire-based investigation designed to assess the perceived diagnostic utility of fluorescence-enhanced three-dimensional (3D) imaging for detecting secondary caries at the margins of direct restorations. The survey evaluated how dental professionals and students of varying clinical experience interpret different imaging modalities and how these interpretations influence diagnostic decision-making. The sample was selected to represent a full spectrum of professional experience, from undergraduate students to experienced clinicians, to assess how experience level affects the interpretation of the image. No objective gold standard, such as histological validation or micro-CT analysis, was performed to confirm the presence or extent of secondary caries in the extracted teeth. This study focused on participants’ subjective interpretation of images and inter-rater agreement rather than on absolute diagnostic accuracy.

### 2.2. Study Population

Participants were recruited using purposive sampling to ensure a diverse range of clinical experience and familiarity with digital imaging. Invitations were distributed via institutional email lists and personal communication. Individuals were eligible to participate if they were either licensed dentists or currently enrolled undergraduate dental students. Those without a formal affiliation to dental education or clinical practice were excluded.

All participants received detailed information about this study and gave informed consent through the online questionnaire platform before participating. The study protocol was approved by the Ethics Committee of the Department of Dentistry, National and Kapodistrian University of Athens, Greece (Approval No. 700/ Approval date: 24 March 2025). It was conducted in accordance with the Declaration of Helsinki and the European General Data Protection Regulation (GDPR). Participation was anonymous and voluntary, and no incentives were offered to respondents.

### 2.3. Image Acquisition

The images used in the questionnaire were obtained from extracted human teeth that already had direct composite resin or amalgam restorations. The collected teeth could be of any tooth type (with or without caries) and were stored refrigerated (−20 °C) immediately after professional tooth cleaning and extraction. Before imaging (photographs and scanning), teeth were left out at room temperature for 2 h. All teeth were scanned by a single trained examiner using a high-resolution intraoral scanner (TRIOS 5, 3Shape A/S, Copenhagen, Denmark) to generate digital 3D models. Fluorescence imaging (to detect bacterial metabolic byproducts indicative of carious activity) was performed using the built-in fluorescence module of the TRIOS 5 intraoral scanner (3Shape, Copenhagen, Denmark). Images were acquired using the manufacturer’s default fluorescence settings (excitation wavelength: ~405 nm), with the standard filter applied for red fluorescence emission detection. All scans were performed under consistent illumination and standardized scanning protocols to ensure reproducibility of fluorescence maps. Each tooth was presented in three formats: a conventional photographic image, a 3D monochromatic model, and a 3D model with fluorescence.

### 2.4. Questionnaire Design

#### A Structured Digital Questionnaire Was Developed Using Microsoft Forms

**Figure 1:**
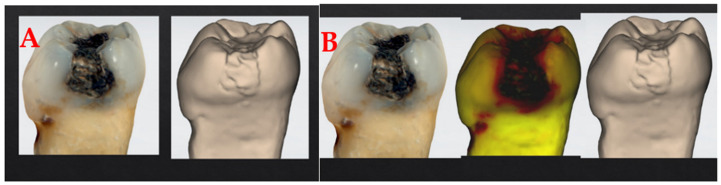
Image 7, when presented in the questionnaire for evaluation, first without a 3D model of fluorescence (**A**) and then with the 3D model of fluorescence (**B**).

To ensure validity, the questionnaire was developed based on the current literature on caries detection and digital diagnostic tools [10]. Draft versions were reviewed by a panel of three experienced dental educators and clinicians to assess clarity and relevance. Formal pilot testing, test–retest reliability, and internal consistency assessments were not conducted, which represents a limitation of the study. The questionnaire was primarily designed to capture subjective perceptions rather than produce psychometric scores. The original instrument was developed in Greek and translated into English using a forward–backward translation procedure. The questionnaire is presented in Appendix A. It consisted of three main parts: (a) Demographic and Professional Information (Age group, Professional status -undergraduate student, dentist, dentist with specialization-, Clinical experience with intraoral scanners), (b) Diagnostic Assessment (Participants were presented with a series of images depicting extracted teeth with direct restorations. Each case was shown in three sequential formats -Conventional photograph, Monochromatic 3D images, 3D images with fluorescence-) (Figure 1) and (c) Perception and Clinical Integration (Participants reported whether they believed fluorescence imaging could enhance clinical practice, their willingness to integrate fluorescence in their diagnostic workflow and barriers to integration (cost, learning curve, accuracy concerns, etc.).

The questionnaire included 29 distinct clinical cases, each represented by extracted human teeth with direct restorations. Each case was presented in three consecutive formats: (1) a conventional photographic image, (2) a monochromatic 3D model, and (3) a 3D model with fluorescence. Therefore, all 29 cases included both fluorescence and non-fluorescence versions, resulting in a total of 87 images presented to each participant. Inclusion criteria consisted of practicing dentists, dental specialists, and postgraduate students enrolled in clinical specialty programs. Postgraduate students were categorized under the ‘dentist with specialization’ group due to their supervised clinical activity within their specialty track

### 2.5. Data Collection

The questionnaire was distributed electronically between March and May 2025 using eForms software (eForms, version 3.2; DataTech Solutions, London, UK; available online). A total of 200 individuals were invited to participate in the study. Each participant completed the survey once, with an average completion time of approximately 10–15 min. Responses were automatically recorded and exported for analysis.

### 2.6. Statistical Analysis

Data was analyzed using IBM SPSS Statistics for Windows, Version 23.0 (IBM Corp., Armonk, NY, USA) [12].

The primary outcome was the perceived diagnostic value of fluorescence-enhanced 3D imaging in identifying secondary caries at restoration margins. Secondary outcomes included differences in diagnostic interpretation across professional experience levels, agreement between groups (Cohen’s kappa), and the influence of visibility/access factors on diagnostic confidence.

Descriptive statistics were computed for demographic variables and response frequencies. A power analysis was conducted for the κ reliability analysis using 29 images rated by 80 participants (40 experienced dentists, 20 minimally experienced dentists, and 20 dentists with no experience). Assuming an expected prevalence of positive ratings of 0.50 and an anticipated κ of 0.50, the standard error of κ was estimated as 0.0148. This yields a test statistic of Z ≈ 33.8, corresponding to power >0.99 for detecting non-zero agreement. Even for lower expected agreement (κ = 0.30), power remains >0.99. Therefore, the sample size of 80 raters provides more than adequate statistical power for the planned inter-rater κ analysis and subgroup comparisons. Chi-square tests were applied to compare categorical variables such as professional experience, experience with 3D imaging, and restoration type with diagnostic responses and perception outcomes. Cohen’s κ was calculated for each image using all available raters. κ was computed separately for two subgroup comparisons: (a) students versus dentists, and (b) participants with versus without experience in 3D imaging. The number of rates per κ estimate ranged from 92 to 94, depending on the number of missing responses. Missing data were treated using pairwise deletion; participants were included in each statistical test only if they had provided a valid response for the variables involved. Kappa values were interpreted according to the Landis and Koch scale [13]: <0 = poor, 0–0.20 = slight, 0.21–0.40 = fair, 0.41–0.60 = moderate, 0.61–0.80 = substantial, 0.81–1.00 = almost perfect agreement. Statistical significance was set at *p* < 0.05.

## 3. Results

**Figure 2:**
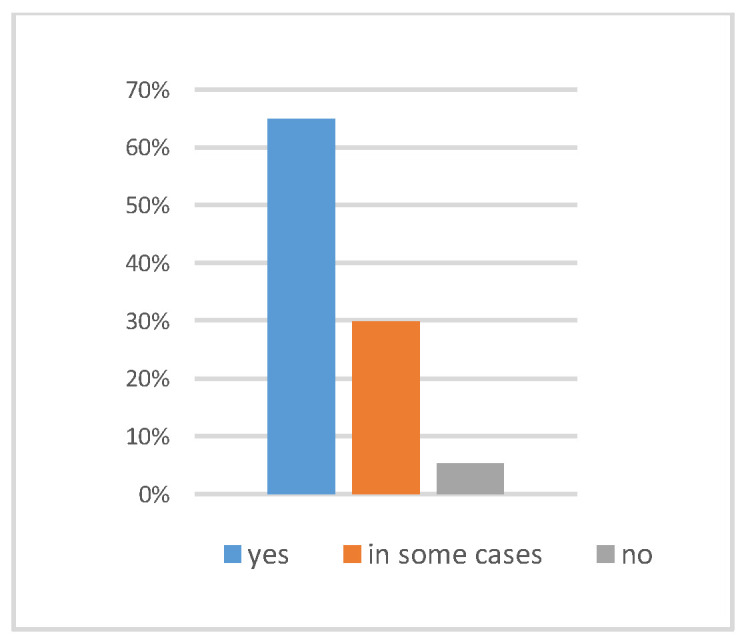
The proportion of respondents reporting that fluorescence images aided their diagnostic decisions. “Yes” indicates the images were helpful, “In some cases” indicates partial usefulness, and “No” indicates no impact on the decision.

A total of 94 participants completed the questionnaire (Response rate 47%). The age distribution was 28.7% (18–24 years), 51.1% (25–41 years), and 20.2% (42–68 years). Regarding prior experience with 3D models, 60.6% reported no experience, 19.1% had less than six months’ experience, and 20.2% reported sufficient familiarity. Of the 94 examined, 64.9% stated that the fluorescence image aided their diagnosis, and 29.8% stated it helped in some cases. (Figure 2)

**Figure 3:**
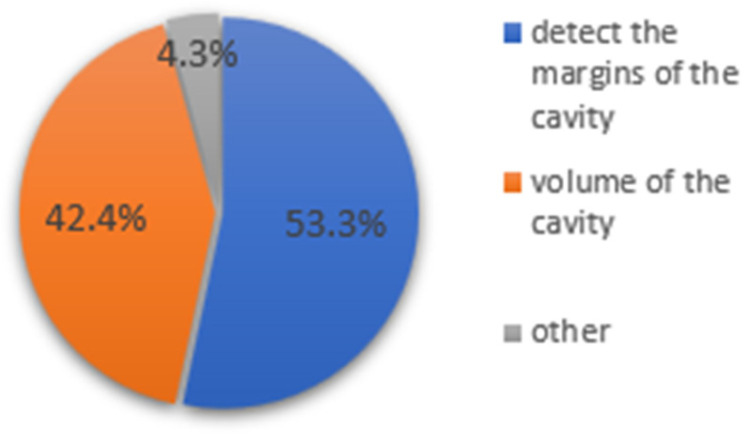
A pie chart that shows the type of help that fluorescence images provided to the tested group.

Out of 92 participants who answered the question “in which part of the diagnostic procedure you were helped the most by fluorescence”, 49 (53.3%) used fluorescence to define the margins of the cavity more clearly, and 39 (42.4%) used fluorescence to detect the volume of the cavity, whereas 4 (4.3%) could not state in what part they were helped (Figure 3).

**Figure 4:**
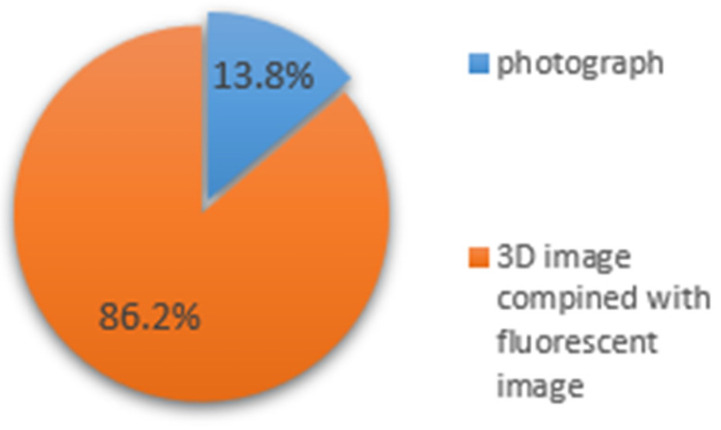
Answers to the question “Which type of presentation do you prefer as more valuable for caries diagnosis?”.

**Figure 5:**
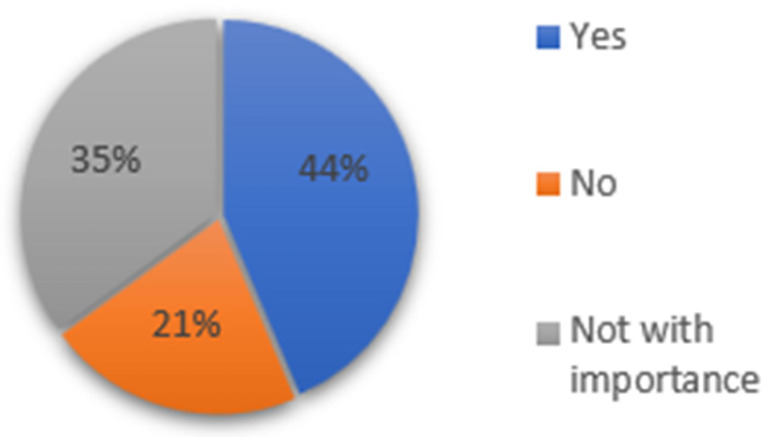
Answers to the question “Did the type of restorations affect your assessment?”.

When comparing image presentation formats, fluorescence-enhanced 3D models were preferred over conventional photographs and monochromatic 3D images (Figure 4). The type of restoration influenced responses: 43.6% reported that restoration type affected diagnostic assessment, with 39.8% finding caries adjacent to composite restorations easier to detect and 26.9% finding amalgam-associated caries easier to identify (Figure 5).

**Figure 6:**
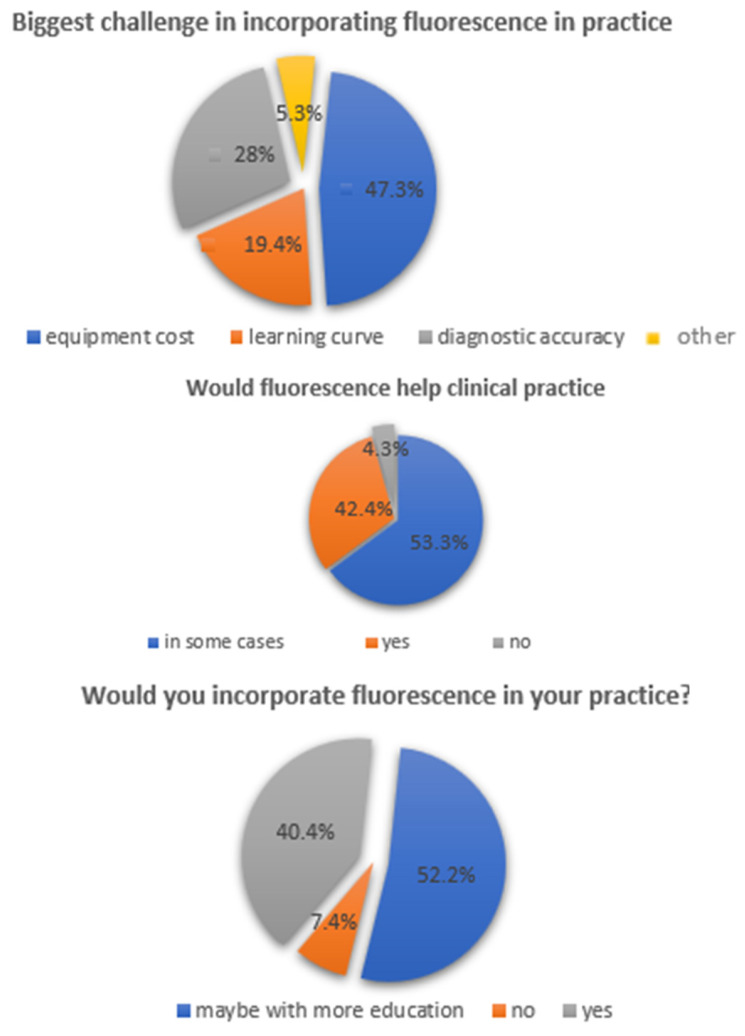
Acceptance of fluorescence images by dentists.

**Figure 7:**
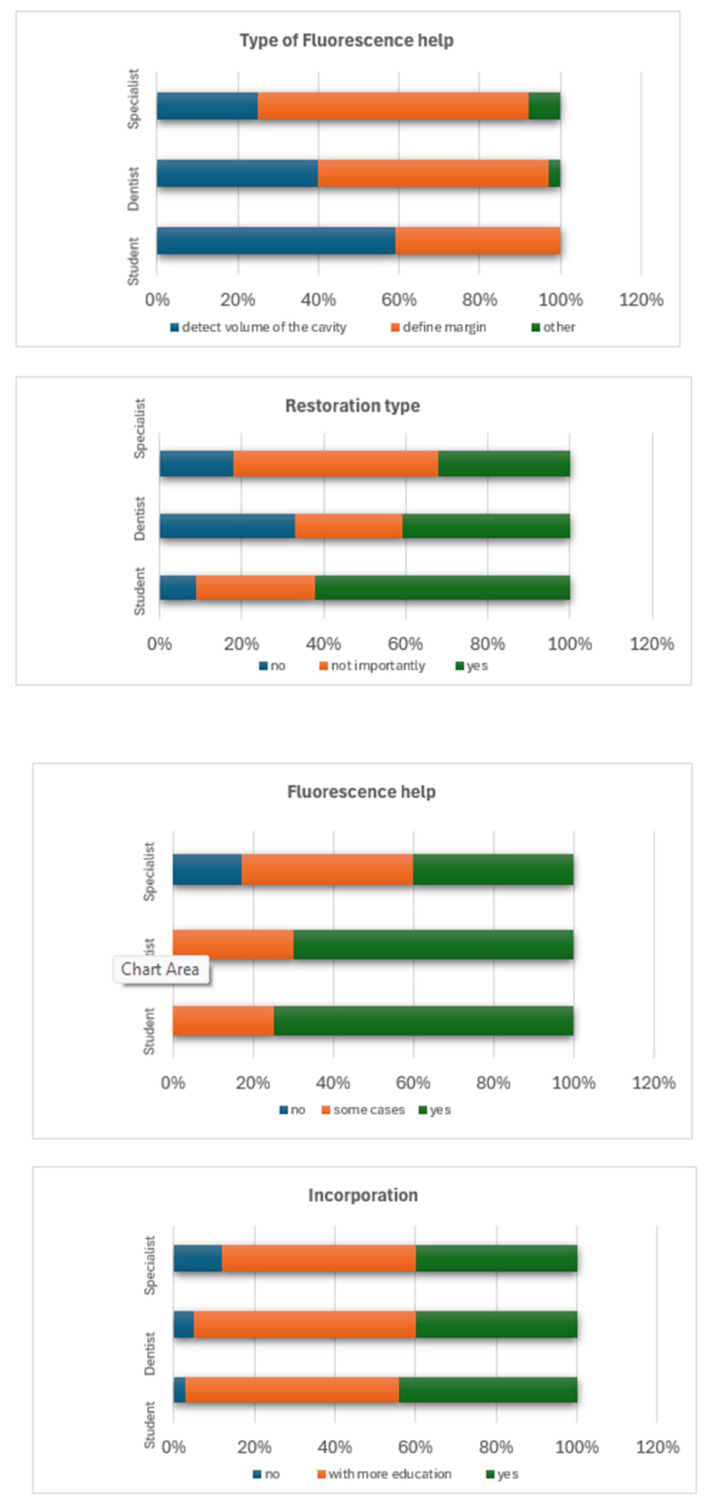

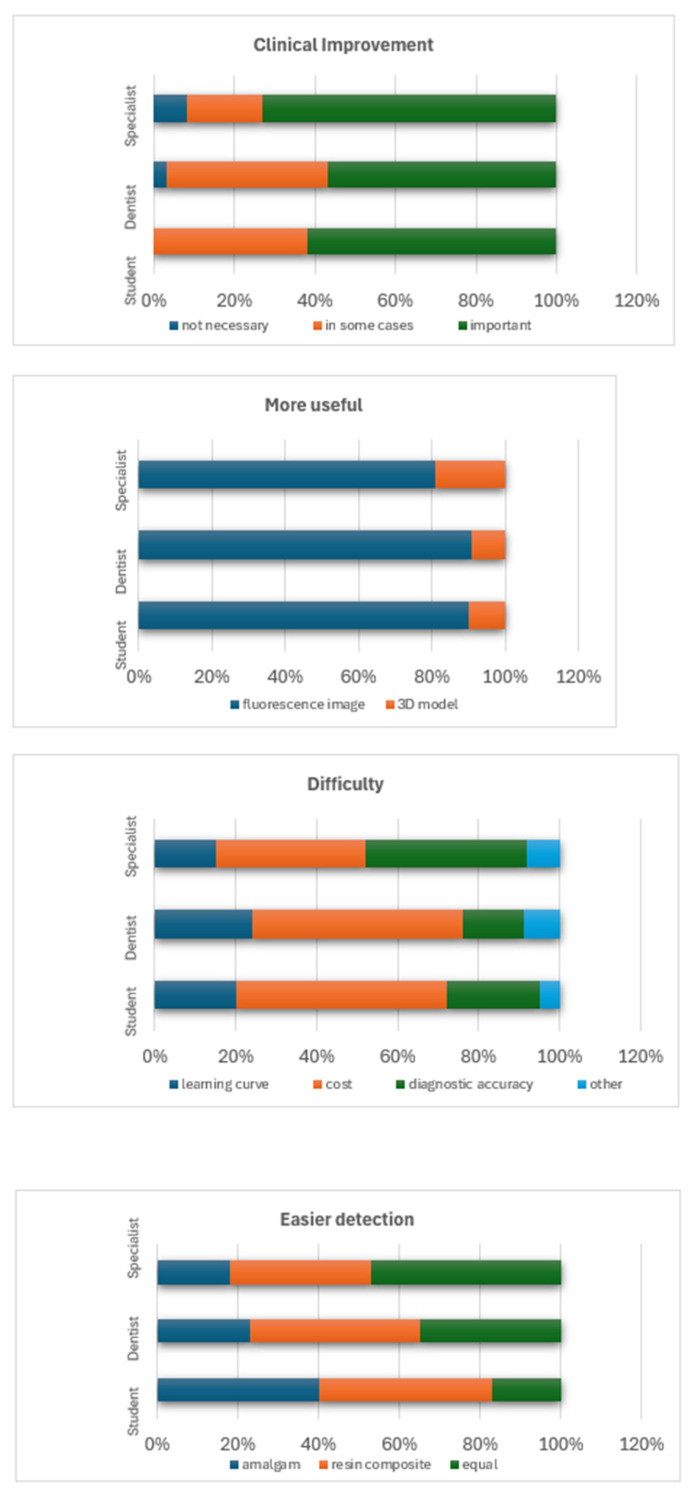
Clustered bar charts were used to illustrate the distribution of responses to the question regarding the profession of the participants.

Regarding clinical integration, most participants expressed willingness to adopt fluorescence imaging, although concerns about cost, learning curve, and accuracy were reported (Figure 6). Figure 7 illustrates differences in perception between undergraduate students and practicing dentists.

**Figure 8:**
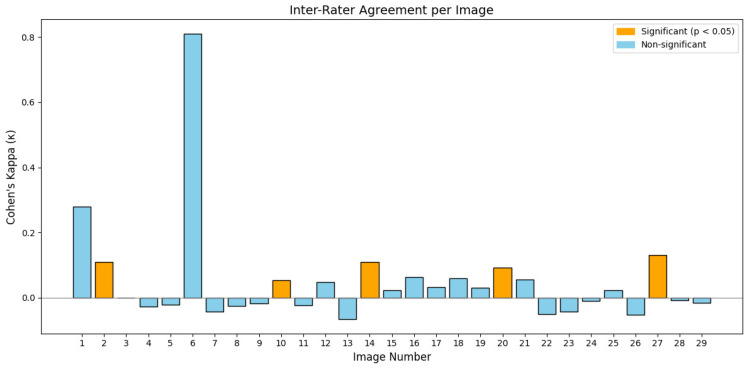
Graphical representation of Cohen’s kappa values per image illustrating inter-rater agreement according to participants’ position.

**Figure 9:**
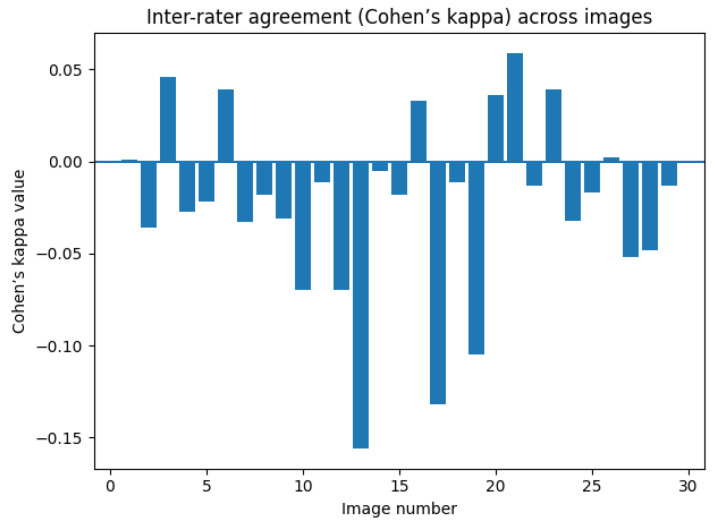
Graphical representation of Cohen’s kappa values per image illustrating inter-rater agreement according to participants’ prior experience with 3D imaging.

Inter-rater agreement across images is summarized numerically in Table 1 and Table 2 and illustrated graphically in Figure 8 and Figure 9, demonstrating predominantly low kappa values across all images. Inter-rater agreement across participants was generally poor. Cohen’s kappa values for most images were close to zero or negative, indicating minimal agreement beyond chance. 5 of 29 images had k values that reached statistical significance, all within the weak agreement range (κ = 0.05–0.13). Only a limited number of images demonstrated weak but statistically significant agreement, and one image showed high κ (0.81) without reaching significance.

## 4. Discussion

The findings of this questionnaire-based study underscore the complexity and subjectivity of diagnosing secondary caries at the margins of direct restorations, even with modern imaging technologies. Despite the integration of fluorescence-enhanced 3D models—designed to improve the visualization of carious lesions, the inter-rater agreement across participants remained generally poor, as reflected by the low or negative Cohen’s kappa values in most cases. Only a limited number of images showed statistically significant agreement, and even then, it was still within the “weak” category. The sole exception, image 6, demonstrated high agreement (κ = 0.810), but this did not reach statistical significance, possibly due to the limited number of consistent raters or image-specific characteristics.

These results align with previous findings in the literature, which highlight that the visual interpretation of carious lesions—especially at restoration margins—is prone to significant observer variation [14,15]. Restoration margins present unique diagnostic challenges due to optical distortions, masking by restorative materials, and anatomical irregularities that hinder consistent evaluation. Subjectivity is further exacerbated in early lesions, where visual–tactile cues are often subtle and nonspecific.

Interestingly, although objective inter-rater agreement was low, most participants reported that fluorescence-enhanced images assisted them in their diagnostic decisions. Most notably, 53.3% found fluorescence helpful for defining cavity margins, while 42.4% used it to assess lesion volume better. When compared with previous studies, these responses align with the underlying optical principles of fluorescence imaging, which highlight metabolic activity and demineralization associated with bacterial invasion [16]. However, the discrepancy between subjective perception and measurable diagnostic consistency suggests that the perceived value of these tools may not directly translate into improved clinical reliability without adequate user training.

An important determinant of perceived benefit appeared to be familiar with digital tools. Participants with prior experience in 3D imaging were more confident in interpreting fluorescence-enhanced images and more open to incorporating such technologies into their diagnostic workflow. This highlights the pivotal role of digital literacy in leveraging advanced diagnostic modalities. Additionally, the restoration material influenced diagnostic participants found it easier to detect caries adjacent to composite restorations than to amalgam restorations, likely due to the greater visual contrast and lower radiographic opacity of composite materials.

Despite general enthusiasm for adopting fluorescence-based diagnostics, several barriers were identified, including concerns about cost, the steep learning curve, and doubts regarding the technique’s reliability. These are consistent with existing studies on the adoption of novel dental technologies, which indicate that limited clinician training, inadequate evidence-based guidelines, and insufficient reimbursement structures often hamper implementation [14,16].

The findings of this study are consistent with those of a recent systematic review, which emphasized that diagnosing secondary caries using fluorescence or near-infrared (NIR) technologies remains a complex and underexplored challenge in contemporary dental practice [17]. The review identified a high risk of bias across many included studies and highlighted wide variability in diagnostic accuracy, with sensitivity and specificity ranging from 18% to 96% [17,18,19]. Notably, fluorescence-based devices often exhibit reduced accuracy near amalgam restorations due to optical interference or staining, leading to frequent false positives. This finding aligns with our observation that clinicians reported greater diagnostic clarity adjacent to composite restorations. Moreover, the systematic review emphasized that examiner variability, lack of standardization, and the diverse properties of restorative materials further compromise consistency, as reflected in our study by the generally poor inter-rater agreement. These insights underscore the urgent need for further in vivo research, diagnostic calibration, and user training to improve the clinical reliability of fluorescence-enhanced imaging [10].

Several limitations of this study must be acknowledged. This study involved a relatively small, institution-specific sample. Although this study was conducted in a controlled setting, the results may not be fully applicable to wider dental populations or diverse international clinical environments. Participants evaluated pre-selected, static images rather than dynamic, interactive 3D models. In real clinical settings, dynamic manipulation of 3D models may enhance diagnostic accuracy. The images represented extracted teeth in an ex vivo setting, which may not accurately reflect the diagnostic complexity of clinical evaluations that involve saliva, patient movement, and clinical context. This study focused on inter-rater agreement rather than diagnostic accuracy against a gold standard such as histological validation or micro-CT. Although this study included participants with different levels of expertise, the impact of specific training on fluorescence imaging interpretation was not evaluated.

In addition, the purposive sampling strategy and the absence of a calculable response rate introduce potential selection bias, as individuals with an existing interest in digital imaging may have been more likely to participate. The use of a single intraoral scanner model and its integrated fluorescence system may also limit the generalizability of the findings, as performance characteristics and visual outputs can vary across devices and manufacturers. Furthermore, although the questionnaire underwent expert review for content clarity, its psychometric properties were not formally validated, and measures such as internal consistency or test–retest reliability were not assessed. These factors should be considered when interpreting the results and planning future research.

Several factors may help explain the discrepancy between participants’ subjective perception that fluorescence enhanced their diagnostic confidence and the objectively low inter-rater agreement. One explanation is the presence of cognitive biases, particularly “technology optimism,” in which clinicians may overestimate the value of novel or visually sophisticated diagnostic tools, independent of their validated diagnostic accuracy [20]. The color-coded fluorescence maps used in this study may create an impression of enhanced clarity even though fluorescence intensity does not always correlate with actual lesion activity and can introduce interpretive artefacts, especially near restorative margins [18]. Three-dimensional visualization may also increase perceived certainty, as clinicians frequently associate advanced imaging formats with improved diagnostic performance, a trend previously observed in studies of digital radiography and 3D dental imaging [21,22]. Furthermore, most participants had limited prior experience with fluorescence interpretation, and insufficient training has been shown to reduce reproducibility and increase susceptibility to misinterpretation in fluorescence-based caries detection [19,23]. These factors likely contributed to the low κ values observed and underscore the need for structured training, calibration, and standardized interpretation protocols when integrating fluorescence imaging into clinical workflows.

For each tooth, images were presented in a fixed order: a conventional photograph, a monochromatic 3D model, and a 3D model with fluorescence. One should acknowledge that this fixed sequence may introduce order effects, as participants’ evaluation of subsequent images could be influenced by their interpretation of the preceding image. Randomization of image order was not performed in this study, which represents a limitation when interpreting the results.

To improve diagnostic consistency and harness the full potential of fluorescence-enhanced 3D imaging, several future directions should be considered. Targeted education and calibration exercises may significantly improve inter-rater agreement. Future studies should investigate how training affects the ability to interpret fluorescence and 3D data accurately. Larger studies involving diverse populations and institutions are needed to confirm the reproducibility of these findings and explore regional or systemic differences in diagnostic behavior. The integration of AI-based diagnostic support systems represents an auspicious direction for future development. AI algorithms trained on large datasets of labeled images could identify carious lesions with greater sensitivity and specificity than unaided human interpretation. Such systems may also serve as educational aids, guiding less experienced clinicians and minimizing inter-operator heterogeneity. Evaluating fluorescence-enhanced 3D imaging in actual clinical settings is essential to determine its feasibility, therapeutic benefits, and patient acceptance. Finally, it should be noted that no objective gold standard was applied to verify the presence or extent of caries in the extracted teeth. Therefore, the study evaluates inter-rater agreement and perceived diagnostic usefulness rather than clinical diagnostic accuracy, which should be considered when interpreting the results.

Given the number of limitations, the findings of this study should be interpreted with caution. Nevertheless, the results provide preliminary insight into how fluorescence-enhanced 3D imaging is perceived by clinicians when evaluating secondary caries at the margins of direct restorations under varying visual access conditions. The study suggests that this imaging modality may have clinical relevance as an adjunctive tool, particularly in situations where conventional visual assessment is challenged. However, further studies with more robust designs, standardized diagnostic criteria, and controlled clinical conditions are required to confirm these observations and to better define the role of fluorescence-enhanced 3D imaging in routine clinical practice.

## 5. Conclusions

Both students and dentists perceived fluorescence-enhanced 3D imaging as a helpful adjunct for identifying secondary caries, particularly for delineating lesion margins and estimating lesion depth. However, these findings reflect subjective impressions derived from an ex vivo, image-based questionnaire rather than actual clinical performance. Diagnostic consistency among participants remained weak, highlighting the inherent variability in visual interpretation and the need for structured calibration and training. While fluorescence-based imaging shows promise as a diagnostic support tool, further in vivo studies, multicenter evaluations, and investigations comparing fluorescence interpretation against validated diagnostic standards are necessary before firm conclusions about its clinical diagnostic accuracy can be drawn. Training, calibration, and integration of Artificial Intelligence (AI) support may enhance clinical reliability. AI algorithms could provide standardized, non-subjective interpretation of fluorescence signals, thereby minimize inter-operator heterogeneity and potentially improve diagnostic consistency across clinicians.

## Figures and Tables

**Table 1:** Inter-rater agreement per image based on Cohen’s Kappa values and statistical significance depending on the position of the participant.

Image	Kappa	Sig
1	0.28	0.488
2 *	0.11 weak	0.011
3	0	0.988
4	−0.027	0.701
5	−0.022	0.633
6	0.81	0.153
7	−0.042	0.380
8	−0.026	0.370
9	−0.018	0.572
10 *	0.053 weak	0.001
11	−0.023	0.609
12	0.047	0.066
13	−0.066	0.131
14 *	0.110 weak	0.044
15	0.022	0.443
16	0.064	0.234
17	0.032	0.527
18	0.060	0.246
19	0.030	0.184
20 *	0.093 weak	0.008
21	0.055	0.373
22	−0.051	0.390
23	−0.042	0.441
24	−0.010	0.701
25	0.023	0.605
26	−0.053	0.280
27 *	0.131 weak	0.004
28	−0.009	0.820
29	−0.015	0.544

* Statistically significant at *p* < 0.05 but κ indicates only weak agreement.

**Table 2:** Inter-rater agreement per image based on Cohen’s kappa values and associated statistical significance, stratified by participants’ prior experience with 3D imaging.

Image	Kappa	Sig
1	0.001	0.937
2	−0.036	0.602
3	0.046	0.311
4	−0.027	0.701
5	−0.022	0.633
6	0.039	0.437
7	−0.033	0.602
8	−0.018	0.391
9	−0.031	0.676
10	−0.070	0.228
11	−0.011	0.847
12	−0.070	0.337
13 *	−0.156 weak	0.004
14	−0.005	0.924
15	−0.018	0.391
16	0.033	0.481
17 *	−0.132 weak	0.020
18	−0.011	0.795
19	−0.105	0.137
20	0.036	0.581
21	0.059	0.140
22	−0.013	0.740
23	0.039	0.377
24	−0.032	0.105
25	−0.017	0.597
26	0.002	0.961
27	−0.052	0.352
28	−0.048	0.145
29	−0.013	0.495

* Statistically significant at *p* < 0.05 but κ indicates only weak agreement.

## Data Availability

The datasets generated and/or analyzed during the current study are available from the corresponding author on reasonable request.

## Appendix A

**Table A1:** The questionnaire used.

Demographic			
Age	18–24	25–40	41–68
Studies	Undergraduate	General Dentist	Dentist with post-postgraduate studies in a clinical field
Experience with the use of intraoral scanner	None	Some (6 months-1 year)	Considerable (more than 1 year)
**Three-dimensional model images (1–15) without fluorescence**			
What do you see in this image? (A) Incipient caries (B) Open cavity (C) No caries			
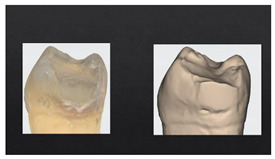
**Three-dimensional model images (1–14) with fluorescence**			
What do you see in this image? (A) Incipient caries (B) Open cavity (C) No caries			
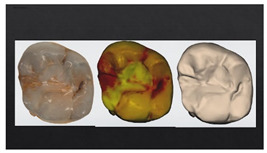
**Dentists’ interpretation**			
Did the presence of the fluorescence image assist in the diagnosis of the lesion?	Yes	No	In some cases
If yes, at which stage of the diagnosis do you believe it contributed the most?	Detection of carious lesion volume	Determine the margins of the cavity	Other
Which form of presentation do you consider most useful for diagnosis?	Photograph	3D model	Three-dimensional model with fluorescence
Did the type of restoration (composite or amalgam) affect your evaluation?	Yes	No	Not significantly
Would you be willing to incorporate fluorescence techniques into your daily practice?	Yes	No	Maybe with training
What do you consider the biggest challenge in implementing the fluorescence technique?	Cost	Learning curve	Diagnostic accuracy
